# Symptoms of Chronic Dysphagia Secondary to Multiple Cervical Inlet Patches and Esophageal Stricture

**DOI:** 10.7759/cureus.33459

**Published:** 2023-01-06

**Authors:** Davong D Phrathep, Stefan Anthony, Kevin D Healey, Ivanna Ward, Michael Herman

**Affiliations:** 1 Family Medicine, Lake Erie College of Osteopathic Medicine, Bradenton, USA; 2 Urology, Lake Erie College of Osteopathic Medicine, Bradenton, USA; 3 Internal Medicine, Philadelphia College of Osteopathic Medicine, Philadelphia, USA; 4 Gastroenterology, Borland Groover, Jacksonville, USA

**Keywords:** endocopic dilation therapy, upper endoscopy, esophageal stricture, progressive dysphagia, cervical inlet patch

## Abstract

Ectopic gastric mucosa can be present throughout the gastrointestinal tract; however, when located within the upper esophagus, it is termed an esophageal inlet patch. To the best of our knowledge, most esophageal inlet patches occur as a single area of gastric mucosa. Here, we present a 44-year-old female who suffered from symptoms of chronic dysphagia and globus sensation for two years due to multiple inlet patches located in the cervical area of the upper esophagus with an associated cervical esophageal stricture. The patient underwent esophageal dilation and proton pump inhibitor therapy, resulting in a resolution of her symptoms. Our case demonstrates the appropriate clinical management of patients suffering from symptoms of chronic dysphagia due to multiple esophageal inlet patches. We recommend routine examination of the cervical esophagus in developing a differential diagnosis of inlet patch, especially in patients with chronic upper dysphagia.

## Introduction

Ectopic gastric mucosa can be present throughout the gastrointestinal tract; however, when located within the upper esophagus, it is termed an esophageal inlet patch and is considered to be a congenital anomaly [1]. Inlet patches are actually found in about 10% of the population [1]. Despite this, they are frequently not diagnosed on endoscopy. It is likely that the underreporting of esophageal inlet patches is related to the fact that they are largely asymptomatic [2]. Complications are directly related to acid secretion from the ectopic gastric mucosa. These complications include, but are not limited to, dyspepsia, reflux, dysphagia, chest pain, esophagitis, ulceration, and stricture [1,2]. Severe complications can occur such as hemorrhage, perforation, and tracheoesophageal fistula [1]. Furthermore, inlet patches can be complicated by *Helicobacter pylori* infection as 73% of patients with concurrent gastric *Helicobacter pylori* will have an infected inlet patch [3]. Diagnosis of an esophageal inlet patch is typically obtained with an upper endoscopy and biopsy. It can also be diagnosed with a barium swallow, pathognomonic with two small indentations along the esophageal wall [1]. Endoscopically, lesions are salmon-colored and velvety with biopsy and histopathology showing a patch gastric mucosa surrounded by normal esophageal mucosa [1]. Treatment depends on the severity of the inlet patch. Asymptomatic patients require no further management; however, those affected may require proton pump inhibitors or dilation depending on symptomatology [1]. Here we present a case of multiple esophageal inlet patches and an associated cervical esophageal stricture causing symptoms of chronic dysphagia in a middle-aged female. 

## Case presentation

**Figure 1:**
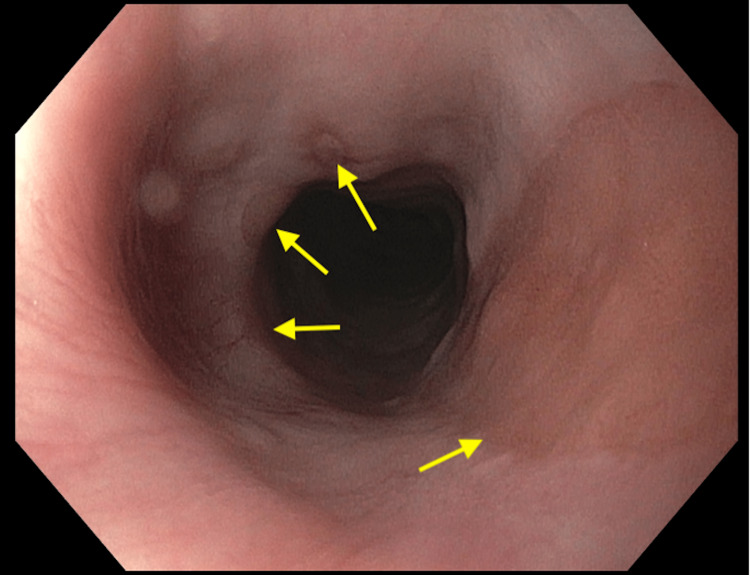
Upper endoscopy reveals multiple salmon-colored mucosae in the upper esophagus.

A 44-year-old white female presented to the clinic with trouble swallowing solid food boluses for the past two years. She reported a recurrent sense of globus sensation and chronic coughing. Dysphagia was reported to occur when the patient ate large solid foods. She stated that there were signs of aspirated food occasionally when eating and associated coughing. The patient had no history of impactions and no symptoms of reflux disease. There was no known personal history of hypertension, diabetes, and allergies, and her family history was unremarkable. The patient reported no alcohol or tobacco use. Vital signs at the clinic showed a temperature of 36.4°C, blood pressure of 138/79 mmHg, respiratory rate of 18/minute, pulse of 66/minute, and O_2_ saturation of 99%. The patient denied nausea, vomiting, hematemesis, hemoptysis, fever, chills, and urinary abnormalities. Physical examination was unremarkable, reporting no signs of epigastric and abdominal pain. The patient's weight was stable. Laboratory data, including a complete blood count and complete metabolic panel, were normal. Due to the patient’s chronic symptoms, an inpatient upper endoscopy was scheduled for the following week. On upper endoscopy, there was multiple salmon-colored mucosae in the upper esophagus (Figure 1).

**Figure 2:**
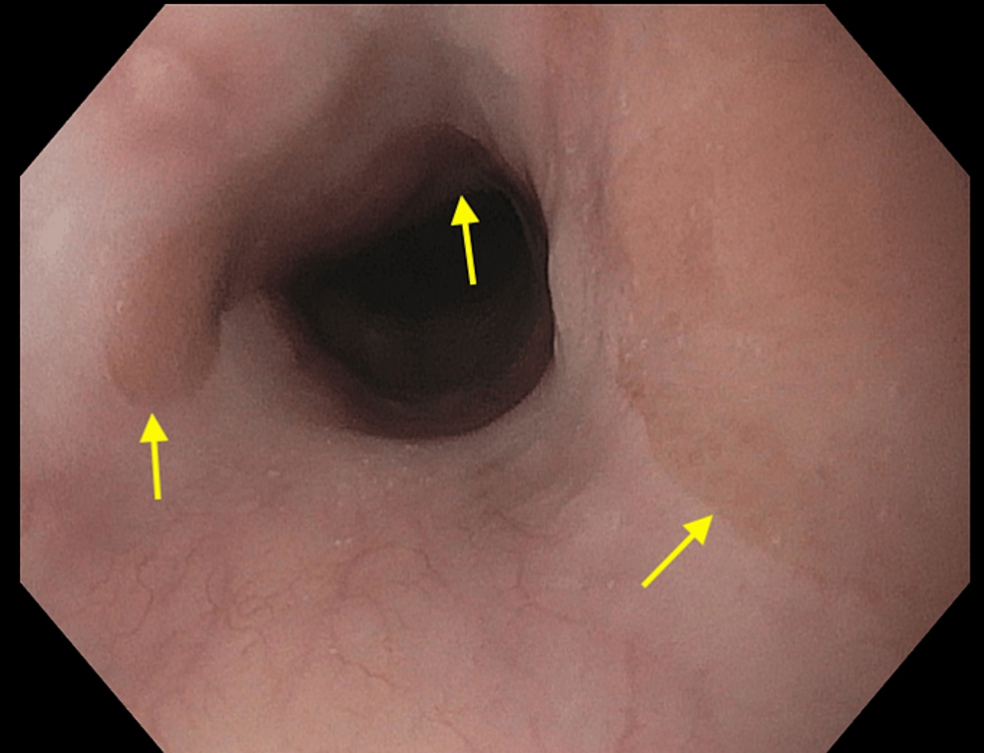
Upper endoscopy demonstrated an associated stricture and additional inlet patches along the upper esophagus.

Additionally, upper endoscopy demonstrated an associated cervical esophageal stricture present (Figure 2).

Multiple biopsies were obtained. Gentle esophageal dilation was completed with a 56 Fr Maloney dilator. The patient was started on 40 mg of omeprazole once daily after the endoscopy. The most likely etiology for the patient’s presentation was related to the multiple inlet patches seen on esophagogastroduodenoscopy. The pathology report was consistent with esophageal inlet patches, revealing multiple areas of gastric mucosa adjacent to the normal esophageal mucosa. Also, the pathology report showed no signs of Barrett’s metaplasia in the distal esophagus and tested negative for *Helicobacter pylori* infection. On follow-up examination four weeks later, the patient reported an improvement and resolution of her symptoms of chronic cough and reflux.

## Discussion

An inlet patch is an anomaly that consists of ectopic gastric mucosa at or beyond the upper esophageal sphincter [1]. Typically, inlet patches are found in the proximal 3 cm of the esophagus just below the upper esophageal sphincter. Current literature has commented on the unclear cause of inlet patches. The most commonly held theory involves the endoderm from the gastric anlage being sequestered or misplaced in the developing esophagus [4]. Another held theory is existing pluripotential cells undergoing local differentiation or metaplasia, resulting in heterotopic gastric mucosa [5]. It has also been hypothesized that the bursting of esophageal retention cysts from occluded esophageal glands results in heterotopic gastric mucosa [6]. The incidence of cervical inlet patches has been reported to be 0.1-10% in adults [7]. Most inlet patches are solitary and affect only part of the esophagus [1]; however, our patient demonstrated multiple inlet patches occurring throughout multiple parts of the esophagus. Although these lesions are usually found incidentally and rarely cause symptoms, our case report highlights multiple inlet patches as the cause of symptoms of chronic dysphagia for two years in a middle-aged female.

To confirm the diagnosis of an inlet patch, endoscopy with biopsy is the typical standard. In our patient, the endoscopy revealed well-demarcated flat, red, velvety patches in the upper esophagus. On biopsy analysis, the typical stratified columnar epithelium of the esophagus was replaced with gastric mucosa in the inlet patch. Inlet patches are often asymptomatic, but when symptoms occur, severe complications can include cervical esophageal strictures [8]. More specifically, esophageal inlet patches can elicit the development of cervical esophageal strictures. It has been hypothesized that cervical esophageal stricture development is the result of parietal cells secreting excess acid in the ectopic gastric mucosa [9]. Inlet patches can be associated with dysphagia due to esophageal rings or webs that cause cervical esophageal strictures in the region of the inlet patch or a function of acid secretion [10]. Standard proton pump inhibitor therapy can be used for symptoms of dysphagia and symptomatic reflux. Histologically, esophageal inlet patches are more commonly characterized by cardiac epithelium rather than oxyntic corpus mucosa [11]. When oxyntic corpus mucosa is discovered, acid production would cause symptoms and proton pump inhibitor therapy would be effective, which rarely occurs [12]. Additionally, gastroesophageal reflux has been suggested to be a major cause of globus sensation [13]. In our patient, symptoms were managed with anti-reflux medications, specifically proton pump inhibitors. Because diagnostic tests for reflux are both invasive and costly, and a negative test does not definitively rule out gastroesophageal reflux [14], the case provided the clinical support to use empirical proton pump inhibitor therapy as a combined intervention of diagnosis and treatment. Therefore, the most likely etiology for the patient’s presentation was related to the multiple inlet patches seen on esophagogastroduodenoscopy.

Although most esophageal inlet patches are asymptomatic, there are different types of management if symptoms do occur. Chronic cough has been associated with inlet patches, due to acid production and irritation of the airways [3,15]. This case demonstrated that the multiple gastric inlet mucosae played a role in excess acid production that caused symptoms and reflux, which improved with proton pump inhibitor use. Patients suffering from cervical esophageal strictures, as seen in our patient, are treated with serial dilation, and multiple biopsies are taken during endoscopy to rule out malignancy [3]. Additionally, radiofrequency ablation (RFA) has been shown to resolve symptoms of sore throat, globus sensation, and cough in patients with esophageal inlet patches [16]. It is suggested that radiofrequency ablation should be performed for larger esophageal inlet patches that affect greater than a third of the circumference of the esophagus [11]. Because relapse of symptoms commonly occurs as a result of residual or recurrence heterotopic gastric mucosa, there have been additional procedures that have been highly effective in symptom management. Endoscopic argon plasma coagulation (APC) has become effective and available for the ablation of esophageal inlet patches. Recent literature identifies APC as an effective therapy for the alleviation of globus sensations and sore throat [17]. However, one limitation of APC involves an increased risk of cervical esophageal stricture development [11]. 

To the best of our knowledge, most esophageal inlet patches occur as a single area of gastric mucosa. The novelty of our case highlights the development of multiple esophageal inlet patches, varying in different sizes, in different areas of the upper esophagus with an associated cervical esophageal stricture. Our report identifies the successful resolution of symptoms with a proton pump inhibitor and dilation. We mention alternative treatment modalities that would’ve been considered if the initial proton pump inhibitor and dilation regimen was ineffective. Endoscopic follow-up and management may be necessary if symptoms are not completely resolved. We suggest that a repeat upper endoscopy should be done for screening of esophageal inlet patches in patients with symptom recurrence or relapse. We recommend that symptomatic patients consider heterotopic gastric mucosa ablation with RFA or APC if proton pump inhibitors and dilation fail to improve symptoms and/or depending on the symptom severity and the size, number, and location of the inlet patches in the esophagus.

## Conclusions

The clinical significance of inlet patches is not well established due to the limited literature; however, patients continue to show symptoms that require timely medical attention. Our case demonstrates an instance where multiple inlet patches in cervical areas of the upper esophagus with an associated cervical esophageal stricture. The patient required immediate clinical attention due to experiencing symptoms of chronic dysphagia and globus sensation for two years. A routine examination of the cervical esophagus is recommended in developing a differential diagnosis of an inlet patch. As an underdiagnosed cervical anomaly, our case highlights the need for consensus guidelines for careful examination, management, and follow-up of inlet patches in patients with symptoms of chronic dysphagia and symptomatic reflux not controlled by standard proton pump inhibitor therapy.
